# The Role of *Prevotella* Species in Female Genital Tract Infections

**DOI:** 10.3390/pathogens13050364

**Published:** 2024-04-28

**Authors:** Sheridan D. George, Olivia T. Van Gerwen, Chaoling Dong, Lúcia G. V. Sousa, Nuno Cerca, Jacob H. Elnaggar, Christopher M. Taylor, Christina A. Muzny

**Affiliations:** 1 Division of Infectious Diseases, Department of Medicine, University of Alabama at Birmingham, Birmingham, AL 35233, USA; oliviavangerwen@uabmc.edu (O.T.V.G.); chaolingdong@uabmc.edu (C.D.); cmuzny@uabmc.edu (C.A.M.); 2 Centre of Biological Engineering (CEB), Laboratory of Research in Biofilms Rosário Oliveira (LIBRO), Campus de Gualtar, University of Minho, 4710-057 Braga, Portugal; luciafilipasousa@gmail.com (L.G.V.S.); nunocerca@ceb.uminho.pt (N.C.); 3 Department of Microbiology, Immunology, and Parasitology, Louisiana State University Health Sciences Center, New Orleans, LA 70112, USA; jelnag@lsuhsc.edu (J.H.E.); ctay15@lsuhsc.edu (C.M.T.)

**Keywords:** *Prevotella*, bacterial vaginosis, endometritis, pelvic inflammatory disease, chorioamnionitis, female genital tract infection

## Abstract

Female genital tract infections (FGTIs) include vaginal infections (e.g., bacterial vaginosis [BV]), endometritis, pelvic inflammatory disease [PID], and chorioamnionitis [amniotic fluid infection]. They commonly occur in women of reproductive age and are strongly associated with multiple adverse health outcomes including increased risk of HIV/sexually transmitted infection acquisition and transmission, infertility, and adverse birth outcomes such as preterm birth. These FGTIs are characterized by a disruption of the cervicovaginal microbiota which largely affects host immunity through the loss of protective, lactic acid-producing *Lactobacillus* spp. and the overgrowth of facultative and strict anaerobic bacteria. *Prevotella* species (spp.), anaerobic Gram-negative rods, are implicated in the pathogenesis of multiple bacterial FGTIs. Specifically, *P. bivia*, *P. amnii*, and *P. timonensis* have unique virulence factors in this setting, including resistance to antibiotics commonly used in treatment. Additionally, evidence suggests that the presence of *Prevotella* spp. in untreated BV cases can lead to infections of the upper female genital tract by ascension into the uterus. This narrative review aims to explore the most common *Prevotella* spp. in FGTIs, highlight their important role in the pathogenesis of FGTIs, and propose future research in this area.

## 1. Introduction

Bacterial female genital tract infections (FGTIs) are characterized by vaginal dysbiosis as a result of an increase in microbial diversity [1]. This is commonly due to the replacement of protective vaginal *Lactobacillus* spp. by facultative and strict anaerobic bacteria [1,2,3]. Bacterial FGTIs include bacterial vaginosis (BV), endometritis, pelvic inflammatory disease (PID), and chorioamnionitis (amniotic fluid infection). They are all common in reproductive-age women (i.e., 12–45 years) [2,4,5] and can lead to multiple adverse health outcomes such as an increased risk of HIV/sexually transmitted infection (STI) acquisition and transmission, infertility, and adverse birth outcomes such as preterm birth [4]. 

BV, the most common vaginal infection, is the most common FGTI, affecting approximately 30% of reproductive-age women [2,6]. Untreated BV can lead to infections of the upper genital tract in women including endometritis, PID, and chorioamnionitis [2,7,8,9]. Furthermore, BV treatment alone incurs an estimated $4.8 billion per year in global healthcare costs, proving FGTIs to be a major global public health concern [6]. 

Endometritis is an inflammation of the endometrium [10] while PID causes the inflammation of the uterus, Fallopian tubes, and/or ovaries [11]. PID commonly occurs in the setting of endometritis, making its prevalence difficult to determine; however, PID is estimated to affect about 8% of women [10,12]. Chorioamnionitis is an infection of the amniotic fluid which occurs in about 4% of deliveries, although its incidence can increase in women with BV and/or STIs [13,14]. The prevalence of these FGTIs can also vary based on race, ethnicity, socioeconomic class, education, and other individual (i.e., sexual behaviors, smoking, etc.) and societal factors (i.e., access to healthcare) [15,16,17,18,19]. 

The vaginal microbiota is the cornerstone of vaginal health, representing a complex and dynamic relationship among many bacterial species (spp.) [20]. *Lactobacillus* spp. are the predominant micro-organisms in most women with an optimal vaginal microbiota, maintaining an acidic environment that is protective against BV and STIs [20]. Fluctuations of the vaginal microbiota can occur during different stages in a woman’s life such as puberty, menses, pregnancy, and menopause [21,22,23]. However, regardless of a woman’s life stage, vaginal dysbiosis can occur [24]. The disruption of the healthy vaginal microbiota is associated with an increased risk of FGTIs with detrimental public health implications [23]. Notably, it is unknown whether anaerobic bacteria cause the loss or displacement of *Lactobacillus* spp. but it is a proposed step in some FGTI etiologies such as BV [25]; other studies have found that *Lactobacillus* phages could also play a role [26,27]. Regardless, the loss or displacement of the protective lactic acid-producing lactobacilli encourages FGTI-associated bacteria, such as *Prevotella* spp., to colonize the vaginal epithelium [28,29].

**Figure 1:**
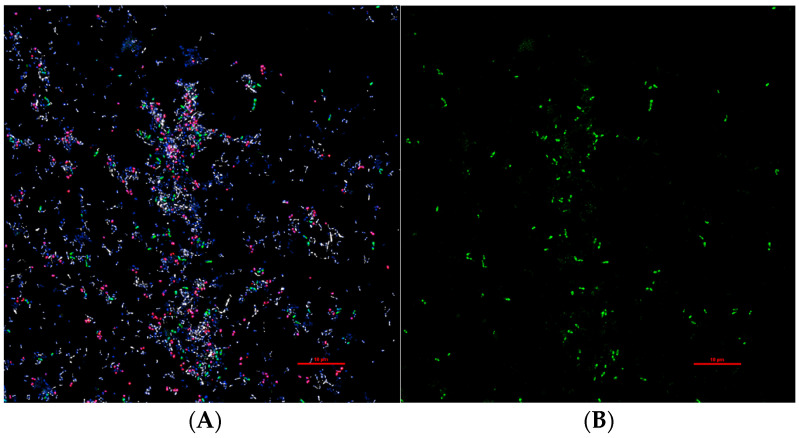
FISH of pure culture bacterial species. (**A**) DAPI, GFP, and TX RED stain featuring three common BV-associated bacteria: *P. bivia* (GFP), *Gardnerella vaginalis* (TX RED), and *Fannyhessea vaginae* (white). (**B**) GFP featuring *P. bivia* in the same culture as (**A**) to highlight its appearance and prevalence within the culture. Images taken at a 60× magnification at high resolution. Figure courtesy of Chaoling Dong, PhD. Abbreviations: peptide nucleic acid fluorescent in situ hybridization (PNA-FISH), 4′,6-diamidino-2-phenylindole (DAPI), green fluorescent protein (GFP), and Texas red (TX RED).

*Prevotella* spp. are an important constituent of the vaginal microbiota in many FGTIs [2,10,21,30]. They are Gram-negative, obligate anaerobes commonly found in the human vaginal microbiota as well as in the gastrointestinal tract, respiratory tract, and oral cavity [31]. Characterized in 1990 from the genus *Bacteroides*, *Prevotella* spp. are differentiated as non-motile, non-spore-forming rods with their color ranging from shiny white to black colonies [32,33,34]. As of 2024, there are over 57 publicly known species of *Prevotella*, most of which are integrated into the human microbiome [33]. For the purposes of this review, only the 48 well-characterized *Prevotella* spp. found in humans will be discussed (Table 1). There are many culture-independent laboratory techniques used to detect *Prevotella* spp. such as quantitative polymerase chain reaction (qPCR), 16S ribosomal ribonucleic acid (16S rRNA) gene sequencing, shotgun metagenomic sequencing, and fluorescent in situ hybridization (FISH) (Figure 1) [35,36].

Exploring the role of *Prevotella* spp. in FGTIs may reveal new mechanisms of BV, endometritis, PID, and chorioamnionitis pathogenesis. The purpose of this narrative review is to discuss the roles of key vaginal *Prevotella* spp. in relation to vaginal dysbiosis, BV, and other FGTIs, discuss the public health implications of infection with these micro-organisms, and propose future research needed to better understand their roles in FGTIs.

## 2. Key Vaginal *Prevotella* spp. in FGTIs

As previously mentioned, *Prevotella* spp. are commonly found in multiple FGTIs [2,49] including BV, endometritis, PID, and chorioamnionitis [2,43,49]. The most prevalent *Prevotella* spp. that can be found in the vaginal microbiota are *P. bivia*, *P. amnii*, and *P. timonensis* [28,38]. These three species are implicated as constituents of FGTIs with varying virulence factors and roles during infection [28,38]. By far, *P. bivia* is the most well-studied and most commonly found *Prevotella* spp. in the female genital tract [68]. 

### 2.1. Prevotella bivia

**Figure 2:**
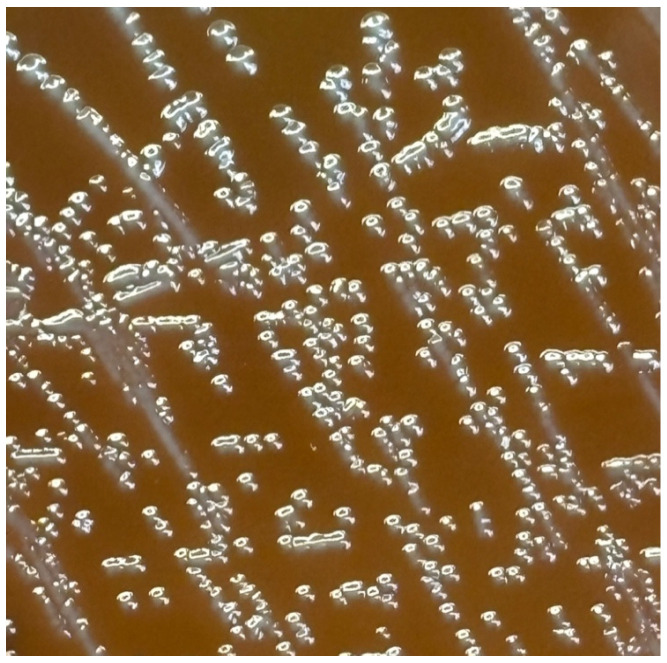
*P. bivia* colonies grown on a blood agar plate after 72 h. Colonies are shiny and gray in color. Figure courtesy of Sheridan D. George.

*P. bivia* is characterized by small, gray colonies which produce sialidase and ammonia (Figure 2, Table 2) [69]. *P. bivia* was first isolated and classified from clinical isolates, many of which originated from the female genital tract such as transabdominal hysterectomy tissue, peritoneal fluid (in a patient with PID), blood from a septic abortion, a cervical-vaginal swab, and vaginal discharge [70]. *P. bivia* is strongly associated with FGTIs, primarily BV, more so than other *Prevotella* spp. [29,71]. *P. bivia* is hypothesized as a key biofilm colonizer early into BV infection, joining the biofilm after *Gardnerella* spp. to create a commensal and persistent relationship [69]. In vitro, *P. bivia* is able to incorporate into *G. vaginalis* biofilms accounting for ~38% of the total biomass [72]. In infected mice, *P. bivia* persisted longer and at a higher density than *G. vaginalis*, despite being inoculated at a lower dosage [73]. In one cohort study, 47% of the women with BV had *P. bivia* present using conventional culture methods [74]. In scanning electron microscopy images, *P. bivia* appears as dispersed biofilms and exhibits crevice colonization during endometrial epithelial tissue infection [28]. Plummer et al. have also suggested that key taxa/spp., particularly *P. bivia*, could have a role in BV recurrence [75]. 

### 2.2. Prevotella amnii

*P. amnii* was first isolated from infected amniotic fluid and is characterized as small, circular, white colonies on blood agar [37]. Of *Prevotella* spp., *P. amnii* has the smallest genome of just 2.37 Mb [33]. *P. amnii* is found in the female reproductive tract and is associated with BV, endometritis, and PID [28,38,39,40]. It can co-occur with *C. trachomatis* infection, and both *P. amnii* and *P. timonensis* have been identified as possible *C. trachomatis* infection biomarkers [33,82]. In one study of women with *C. trachomatis* infection, the prevalence of *P. amnii* was 3.2% while in women without *C. trachomatis*, its prevalence was much lower (0.001%) [82]. In another study of women at risk for chlamydial cervicitis, *P. amnii* was found in 59% of the women who subsequently developed PID compared to 24% of the women who did not [38]. In a spontaneous preterm birth study, *P. amnii* was found to be significantly associated with preterm birth, while *P. bivia*, *P. timonensis*, and other *Prevotella* spp. were not [83]. Although *P. amnii* is understudied, its presence could signify adverse sequelae although more research is required to fully understand its role in this setting.

### 2.3. Prevotella timonensis

*P. timonensis*, originally isolated from a human breast abscess [76], is another abundant *Prevotella* spp. in FGTIs. While other *Prevotella* spp. can be found in women with and without BV (i.e., *P. bivia*, etc. [20]), *P. timonensis* is primarily only found in women with BV [84]. In a previous 16S rRNA sequencing study, *P. timonensis* has been found in 76% of the women with BV compared to only 9% of the women without BV [72]. Growth on blood agar is similar to both *P. bivia* and *P. amnii* but with slightly larger colonies of 1–2 mm (Table 2) [76]. Similar to *P. amnii*, *P. timonensis* can also co-occur in *C. trachomatis* infections [33,82]. *P. timonensis* is also associated with the persistence and slower regression of cervical intraepithelial neoplasia in women with high-risk human papillomavirus (HPV) subtypes [33,85].

## 3. Virulence Factors of Vaginal *Prevotella* spp.

*Prevotella* spp. were first identified to be dark-colored colonies with a moderate potential to break down carbohydrates with bile sensitivity [32]. Now, much more is known about their physiology, especially their virulence pathways and the mechanisms of action during infection. *Prevotella* spp. are known to have adhesins, fimbriae, and hemolysins and secrete nucleases, proteases, lipopolysaccharides (LPS), exopolysaccharides, and hydrolases [77,86,87]. Each of these virulence factors can lead to biofilm formation and antibiotic resistance [77]. 

During FGTIs, *Prevotella* spp. can secrete multiple products, such as hydrolases and ammonia, which can contribute to an increase in virulence [77,87]. Of these secreted hydrolases, sialidase, also known as neuraminidase, is a common enzyme secreted by *Prevotella* spp. (Table 3) [28,80,88]. Sialidase acts by degrading immunoglobins and mucins of host epithelial cells [86,88]. This degradation catalyzes sialic acids and allows bacterial adhesion to host epithelial cells, resulting in reduced immunity to pathogens [89]. In the vagina, sialidases promote the breakdown of the protective mucus layer, which leads to bacterial attachment and the release of carbon sources to facilitate bacterial growth [73,90]. *G. vaginalis*, a key BV-associated bacterium, is most known for its sialidase activity and adherence, but it is important to note that *Prevotella* spp. secrete sialidase as well [33,87]. Ammonia production is also a dominant characteristic of *Prevotella* spp., notably *P. bivia*, which has been found to support *G. vaginalis* growth in vitro [69,78]. *P. bivia* commonly appears and persists during FGTIs likely due to its resistance against antibiotics used to treat these infections [75,77]. Metronidazole, a common treatment for anaerobic bacterial infections in FGTIs, inhibits protein synthesis [91]. Three clinical metronidazole-resistant *P. bivia* strains have been found to harbor a mobile genetic element, encoding a novel nim gene, nimK, and a small efflux MDR transporter [81]. Although the presence of the nimK gene and the MDR transporter across multiple *P. bivia* isolates beyond the three isolates evaluated in this study is not yet known, *P. bivia* resistance to metronidazole could contribute to persistent infections, which may facilitate *P. bivia* ascension into the upper female genital tract [28,79,91]. 

Other *Prevotella* spp. beyond *P. bivia* also possess virulence factors. Previous 3D endometrial epithelial model studies have shown that *P. amnii*, *P. buccae*, *P. corporis*, *P. denticola*, *P. disiens*, *P. histicola*, and *P. timonensis* all produce sialidase, although *P. timonensis* produces more compared to the other species [28]. *P. timonensis* also had elongated microvilli wrapped around the surface of the 3D model, the greatest number of mucin degradation pathways, and interactions with vaginal dendritic cells which promote inflammation [28,33]. To date, these factors have not been shown in any other *Prevotella* spp. [28,33,77]. In this same endometrial epithelial model, *P. disiens* was significantly more cytotoxic to endometrial epithelial cells than other *Prevotella* spp., confirming its significant pathogenicity [28]. Many vaginal *Prevotella* spp. including *P. bivia*, *P. amnii*., and *P. timonensis* harbor clindamycin antibiotic resistance [46,79]. Each of these virulence mechanisms of *Prevotella* spp. could play key roles in the pathogenesis of FGTIs.

## 4. Role of *Prevotella* spp. in the Pathogenesis of BV

BV is characterized by the formation of a polymicrobial biofilm on vaginal epithelial cells [90]. Despite decades of extensive research, the etiology of BV remains controversial [92]. This has directly impacted improvements in the diagnosis, treatment, and prevention of this common vaginal infection [75,93]. With BV affecting up to 30% of reproductive-age women, a better understanding of its etiology will give critical insights into its management [7]. *Prevotella* spp. are commonly found in women with BV but their exact role in its pathogenesis remains unknown [94]. *Prevotella* spp. are part of the healthy vaginal microbiota, albeit in low numbers; however, their overgrowth is correlated with BV along with other anaerobic bacteria such as *G. vaginalis* and *F. vaginae* [29,92,94,95]. In a cohort of non-pregnant women with BV, *Prevotella* spp. represented 44% of all anaerobes isolated, suggesting their importance in BV infection [74].

In a study based on women who have sex with women (WSW), the mean relative abundance of *P. bivia* and *G. vaginalis* became sequentially higher 4 days (for *P. bivia*) and 3 days (for *G. vaginalis*) prior to the development of incident BV (iBV). This is compared to women who maintained normal vaginal microbiota during the study [71]. Based partly on the WSW study performed by Muzny et al., a conceptual model of BV pathogenesis was developed associating *P. bivia* and *G. vaginalis* with BV infection [69]. In this model, it is proposed that after the initial adhesion and displacement of vaginal lactobacilli by *G. vaginalis*, *P. bivia* attaches to the developing BV biofilm, prospers using amino acids produced by *G. vaginalis* for growth, and secretes ammonia [69,78]. The ammonia secreted by *P. bivia* enhances *G. vaginalis* growth, causing the biofilm to flourish [69,78]. The combined sialidase production of *G. vaginalis* and *P. bivia* breaks down the mucous layer and establishes an adherent biofilm [69]. This symbiotic relationship is thought to be essential in BV biofilm infection, adherence, and persistence [69,78].

In mouse models, *P. bivia* produced significant levels of sialidase during BV co-infection with *G. vaginalis* and alone, in vitro and in vivo [73]. Interestingly, *P. bivia* can colonize mice without *G. vaginalis*, even when inoculated in low doses [73]. In these mouse models, *G. vaginalis* co-cultured with *P. bivia* enhanced ascending uterine infection and invasion, supportive of an important role in the pathogenesis of upper FGTIs, such as endometritis and PID [49,73,96]. However, mice maintain a vaginal pH closer to seven and do not have a *Lactobacillus* spp. dominant vaginal microbiota like humans, which suggests that further studies are required to characterize this phenomenon in humans [73]. 

As previously mentioned, non-*P. bivia* spp. such as *P. amnii*, *P. timonensis*, *P. corporis*, and *P. buccalis* can also be found in women with BV [33]. There is mostly a genus-level focus on less prevalent *Prevotella* spp. sialidase and ammonia production, making it difficult to understand individual species’ involvement in biofilm formation. A recent study concluded that a higher relative abundance of *Prevotella* spp. remaining after BV treatment resulted in a higher likelihood of BV reoccurrence compared to women with a lower *Prevotella* spp. abundance [75]. These new data propose a potentially important role of *Prevotella* spp. in recurrent BV and high persistence rates even after treatment [75]. Untreated BV can lead to upper FGTIs such as endometritis and PID, suggesting why many *Prevotella* spp. are found in both lower and upper FGTIs (Table 1) [73].

## 5. Role of *Prevotella* spp. in the Pathogenesis of Endometritis and PID

Endometritis commonly co-occurs with PID, making it difficult to differentiate between the two conditions. Each can have a similar polymicrobial species composition including BVAB (BV-associated bacteria), *Ureaplasma* spp., *Mycoplasma genitalium*, etc. [10,97]. Because of this co-occurrence, endometritis is clinically underdiagnosed and statistically underrepresented in clinical and research settings [10,98]. Endometritis and PID have been closely associated with *C. trachomatis* and *N. gonorrhoeae* infections; however, studies have shown that at least half of the combined PID and endometritis cases have no trace of these bacterial STIs [99,100]. Interestingly, non-gonococcal, non-chlamydial PID caused by BVAB, such as *Prevotella* spp., is more prevalent than gonococcal/chlamydial PID, based on available data [49,101,102]. In a study of 545 participants with suspected PID, those with BVAB were more likely to have endometritis and recurrent PID than those without [97]. In another study of 278 women, those with acute endometritis were more likely to be infected with Gram-negative rods, such as *P. bivia*, along with having clinically diagnosed BV [49]. It is suggested that *P. bivia* could ascend from the vagina to the uterine cavity, causing endometritis and PID [103]. Most importantly, BVAB have been implicated in causing endometritis and PID individually from *C. trachomatis* or *N. gonorrhoeae* or any other FGTI-associated bacterial species [49]. 

As most women with PID are treated with antibiotics targeting chlamydia and/or gonorrhea, it has been suggested that a more effective treatment approach should also consider BVAB [49]. Petrina et al. tested endometrial biopsy isolates from women with PID and histologically confirmed endometritis cases with different antibiotic treatments after extraction in vitro [46]. After treatment with ceftriaxone, the only species remaining in the isolates were *Prevotella* spp. *Prevotella* spp. were susceptible to metronidazole while over half of the *G. vaginalis* present in the isolates were resistant, suggesting that several antibiotics could be required to clear endometritis and PID. Having notable resistance to ceftriaxone in vitro, *Prevotella* spp. could influence endometritis and PID case persistence and recurrence [46]. In another study testing different antibiotic efficacies for PID treatment, women with confirmed cases of PID were given common antibiotics prescribed for PID and chlamydia/gonorrhea (ceftriaxone and doxycycline) combined with metronidazole to compare to women given the same ceftriaxone and doxycycline treatment plus a placebo [104]. The women given ceftriaxone, doxycycline, and metronidazole had fewer endometrial anaerobic organisms and less pelvic discomfort than the placebo group at 1 month after treatment [104]. Because metronidazole is the recommended treatment for BV, this suggests that anaerobic bacteria (including *Prevotella* spp.) might also be playing a major role in PID cases [104]. This discovery has opened new questions in endometritis/PID research as well as a need to explore the role of *Prevotella* spp. in acute and chronic non-gonococcal, non-chlamydial endometritis/PID cases. 

Of the many bacterial species implicated in PID, *Prevotella* spp. are found in half of the women with PID [46]. Of the three types of bacterial groups of PID, BVAB are associated with a 2-fold increased risk of PID [101,102]. In a study of women at risk for chlamydial cervicitis, the women with *P. amnii* were at an elevated risk of developing subsequent PID [38]. 16S rRNA samples from these same women also demonstrated that *P. timonensis* was the most prevalent bacterial species collected prior to PID development [38]. These data suggest that *P. amnii* and *P. timonensis* may be associated with an elevated risk of PID among high-risk women [38]. In addition, chronic PID can result in tubo-ovarian abscesses where *Prevotella* spp. predominate [105]. *Prevotella* spp. significantly contribute to the development of PID in women with and without STIs [38,49]. BVAB, particularly *P. amnii* and *P. timonensis*, are strongly associated with an increased risk of PID, regardless of the presence of STIs [38,49]. 

Because of the close association between endometritis and PID, additional research is necessary in women with endometritis alone as well as *Prevotella* spp.-specific involvement in this infection. Although there are difficulties in differentiating the cases of endometritis and PID, both conditions have a strong presence of BVAB including *Prevotella* spp. [46,104].

## 6. Role of *Prevotella* spp. in the Pathogenesis of Chorioamnionitis 

Chorioamnionitis is a polymicrobial FGTI involving amniotic fluid during pregnancy or after delivery [13]. It is associated with multiple adverse health outcomes such as preterm birth, impaired infant brain development, chronic lung disease in infants, and, if left untreated, both maternal and infant mortality [13]. *Prevotella* spp., typically reported at the genus level, have been implicated in chorioamnionitis [106]. *P. bivia* is noted to be found in more serious cases of chorioamnionitis with high-grade inflammation and fetal vasculitis [43,96] and is associated with an increased risk of preterm birth [106,107]. *P. bivia* is found twice as often in severe cases of chorioamnionitis than in moderate cases [43], but its exact role is unknown. Other *Prevotella* spp. have not been specifically noted in women with chorioamnionitis to date; however, not many studies have explored this connection. More investigation is required to examine the relationship between *Prevotella* spp. and chorioamnionitis.

## 7. Future Areas of Research

Many FGTIs are dynamic, polymicrobial infections with complex etiologies. Many bacterial species have important roles in FGTIs, but *Prevotella* spp. possibly have the most questions unanswered. Because many FGTIs remain controversial in their exact etiology and pathophysiology, the knowledge gaps and research opportunities are plentiful in the field. The ultimate question of BV etiology still remains. Because BVAB are found in endometritis, PID, chorioamnionitis as well as other FGTIs, discovering the precise etiology of BV is key to treating and preventing many FGTIs. Also, the mechanism of how BV could cause other FGTIs like endometritis and PID is not fully understood or confirmed in humans. BV-associated endometritis, PID, and chorioamnionitis cases will likely remain a mystery until BV etiology is elucidated. Similarly, the exact role of *Prevotella* spp. in FGTIs is yet to be understood. 

Many studies characterize *Prevotella* at the genus level only, leaving specific species involvement up for debate. *P. bivia* has been widely studied within the FGTI field, but *P. amnii* and *P. timonensis* deserve further attention. Ultimately, most *Prevotella* spp. require more attention to understand their roles in FGTIs as many appear at least in small quantities [38,49,83]. The interaction between BV, endometritis, PID, and chorioamnionitis infections requires more research on the specific mechanism of action of the bacterial species involved. More attention to *Prevotella* spp. in chorioamnionitis infection is needed, especially to investigate *Prevotella* spp. other than *P. bivia*. Current data suggest that *Prevotella* spp. influence complex relationships and bacterial ascension up the female genital tract to create environments for FGTIs to flourish [33,69,73].

## 8. Conclusions

*Prevotella* spp. are prevalent in FGTIs, notably *P. bivia*, *P. amnii*, and *P. timonensis*. BV, endometritis, PID, and chorioamnionitis all propose the importance of *Prevotella* spp. in their etiologies and the importance of focusing future investigations on *Prevotella* spp. during FGTIs. Critical gaps remain in the specifics of *Prevotella* spp. pathogenesis during infection. The unique virulence factors harbored by *Prevotella* spp. increase their ability to persist during infections, even after the use of certain antibiotics. FGTI treatment methods by testing the antibiotic resistance of BVAB isolates, such as *Prevotella* spp., could be necessary in recurrent and persistent FGTI cases. Advancing our understanding of the role of *Prevotella* spp. in FGTIs will improve diagnostic accuracy, treatment efficacy, and women’s health outcomes.

## Figures and Tables

**Table 1:** *Prevotella* spp. associated with human hosts and their respective organ systems. Listed are the most common/relevant clinical syndromes and organ system involvements as *Prevotella* spp. are found in many infections.

Species Name	Primary Clinical Syndrome	Common Organ System Involvement
*P. amnii*	BV, Endometritis, PID	Vagina, Amniotic fluid [37,38,39,40]
*P. aurantiaca*	Periodontal disease	Oral [41]
*P. baroniae*	Oral disease(s) ^1^	Oral, Brain abscess [41]
*P. bergensis*	Gut dysbiosis/Unknown	Oral, Breast abscess, Skin/soft tissue, Gut [41,42]
*P. bivia*	BV, Endometritis, PID, Chorioamnionitis	Vagina, Skin/soft tissue [33,43,44]
*P. brunnea*	Unknown	Skin/soft tissue [45]
*P. buccae*	Oral disease(s) ^1^	Oral [41]
*P. buccalis*	Oral disease(s) ^1^, BV, Endometritis, PID	Vagina, Oral, Gut [33,41,46]
*P. colorans*	Unknown	Skin/soft tissue [45]
*P. copri*	Gut dysbiosis, Rheumatoid arthritis	Gut [31]
*P. corporis*	BV	Vagina, Oral, Gut [33,47]
*P. dentalis*	Oral disease(s) ^1^	Oral [41]
*P. denticola*	Oral disease(s) ^1^, BV, Endometritis, PID	Vagina, Oral [28,41,46]
*P. disiens*	BV, Endometritis, PID, Gingivitis	Vagina, Gut, Bartholin abscess, Oral [28,33,48,49]
*P. enoeca*	Periodontal disease	Oral [41]
*P. fusca*	Periodontal disease	Oral [41]
*P. heparinolytica*	Periodontal abscesses	Oral, Brain abscess [41]
*P. histicola*	Oral disease(s) ^1^	Vagina, Oral, Gut, Airways [33,41,50,51]
*P. ihumii*	Gut microbiome/Unknown	Gut [52]
*P. intermedia*	Oral disease(s) ^1^	Oral, Empyema, Gut [33,41,53]
*P. jejuni*	Gut dysbiosis/Unknown	Gut, Oral [41]
*P. koreensis*	Oral Disease(s) ^1^, Gut dysbiosis	Oral, Gut [42,54]
*P. lascolaii*	BV	Vagina, Gut [42,55]
*P. loescheii*	Oral disease(s) ^1^, BV	Oral, Gut, Skin/soft tissue [41,56,57]
*P. maculosa*	Oral disease(s) ^1^	Oral [41]
*P. marseillensis*	Gut dysbiosis/Unknown	Gut [58]
*P. marshii*	Oral disease(s) ^1^	Oral [41]
*P. massilia timonensis*	Gut microbiome/Unknown	Gut [59]
*P. melaninogenica*	Oral disease(s) ^1^, BV, Endometritis, PID	Vagina, Oral, Sputum, Gut [33,41,60]
*P. micans*	Peri-implantitis	Oral, Gut [41,61]
*P. multiformis*	Oral disease(s) ^1^	Oral, Gut [41,45]
*P. multisaccharivorax*	Oral disease(s) ^1^	Oral [41]
*P. nanceiensis*	Unknown	Blood, Oral, Airways, Gut [45]
*P. nigrescens*	Oral disease(s) ^1^	Oral, Gut [31,45]
*P. oralis*	Oral disease(s) ^1^, BV, PID	Vagina, Gut, Oral [30,41,42,47]
*P. oris*	Oral disease(s) ^1^	Oral, Airways, Gut [41,45]
*P. oulorum*	Oral disease(s) ^1^	Oral [41]
*P. pallens*	Oral disease(s) ^1^	Oral, Gut [41,45]
*P. phocaeensis*	Gut dysbiosis	Gut [62]
*P. pleuritidis*	Pleuritis	Pleural fluid, Lung abscess [63,64]
*P. rara*	Gut microbiome/Unknown	Gut [65]
*P. saccharolytica*	Peri-implant mucositis	Oral [41]
*P. salivae*	Oral disease(s) ^1^, Gut dysbiosis	Oral, Gut [41,66]
*P. scopos*	Oral disease(s) ^1^	Oral [41]
*P. shahii*	Oral disease(s) ^1^	Oral [41]
*P. stercorea*	Gut microbiome/dysbiosis	Gut [31]
*P. timonensis*	BV, Endometritis, PID	Vagina, Breast abscess [38,46,67]
*P. veroralis*	Oral disease(s) ^1^	Oral, Gut, Airways [41,45]

^1^ Oral diseases include, but are not limited to periodontitis, periodontal disease, periodontal abscess, endodontic infection, dental caries, halitosis, and peri-implantitis [41]. Oral *Prevotella* spp. may appear in FGTIs depending upon a woman’s sexual activities [33]. Abbreviations: BV: bacterial vaginosis, and PID: pelvic inflammatory disease.

**Table 2:** Morphological and physiological differences between key *Prevotella* spp. involved in FGTIs.

Characteristic	*P. bivia*	*P. amnii*	P. timonensis
Genomic length (Mb)	2.49 ± 0.08 [33]	2.4 ± 0.03 [33]	3.09 ± 0.16 [33]
Colony appearance	Circular, gray, shiny, circular colonies (Figure 2)	Circular, white, smooth, shiny, circular colonies [37]	Circular, light gray, smooth, shiny colonies [76]
Colony size	<1 mm (Figure 2)	<1 mm [37]	1–2 mm [76]
Virulence factors	Sialidase, ammonia, mucin expression, antibiotic resistance [28,69,77,78]	Antibiotic resistance, sialidase [79,80]	Dramatic sialidase secretion, mucin expression, antibiotic resistance [28,79]
Biofilm appearance in vivo	Dispersed biofilm, crevice colonization [28]	Unknown	Elongated microvilli, dispersed biofilm [28]
FGTI	BV, Endometritis, PID, Chorioamnionitis	BV, Endometritis, PID	BV, Endometritis, PID
Antibiotic resistance	Yes (Clindamycin, Ceftriaxone, Metronidazole) [79,81]	Yes (Clindamycin) [79]	Yes (Clindamycin) [79]

Abbreviations: BV: bacterial vaginosis; PID: pelvic inflammatory disease.

**Table 3:** *Prevotella* spp. virulence factors in vitro, in mice models, and in humans.

Prevotella Species	In vitro	Mice	Humans
*P. bivia*	Antibiotic resistance, sialidase, ammonia, cell crevice colonization [28,78,79]	Sialidase, ascension [73]	Sialidase, ammonia [57], antibiotic resistance [81]
*P. amnii*	Antibiotic resistance [79]	Unknown	Sialidase [80]
*P. timonensis*	Antibiotic resistance [79], elongated microvilli, mucin activity, dramatic sialidase secretion, biofilm dispersion [28]	Unknown	Sialidase [80]

## Data Availability

Not applicable.
